# A Study Protocol to Assess the Association Between Ambient Air Pollution and Asthma and Other Respiratory Health Outcomes Amongst Children Below 5 Years of Age in Alexandra Township’s Early Childhood Development Centers, Johannesburg

**DOI:** 10.3390/mps8040084

**Published:** 2025-08-01

**Authors:** Velisha Thompson, Joyce Shirinde, Masilu D. Masekameni, Thokozani P. Mbonane

**Affiliations:** 1 Department of Environmental Health, Faculty of Health Sciences, University of Johannesburg, Johannesburg 2001, South Africamaseksd@unisa.ac.za (M.D.M.); 2 School of Health Systems and Public Health, Health Sciences Faculty, University of Pretoria, P.O. Box 667, Pretoria 0001, South Africa; joyce.shirinde@up.ac.za; 3 Developmental Studies, School of Social Sciences, University of South Africa, Pretoria 0001, South Africa

**Keywords:** asthma, respiratory condition, air pollution, environmental exposure, health risk assessment

## Abstract

Air pollution is linked to childhood mortality and morbidity in low- and middle-income countries globally. There is growing evidence linking air pollution to asthma and other respiratory diseases in children. Studies have shown that children are likely to experience asthma due to their narrow airways and their heightened sensitivity to environmental irritants. This study aims to investigate the relationship between ambient air pollution and respiratory diseases in children under the age of 5. The study will be conducted in the informal township of Alexandra, north of Johannesburg, South Africa. A quantitative approach will be used in this cross-sectional analytical study. Data will be collected using different tools that include a questionnaire to determine the prevalence of asthma and respiratory disease and potential risk factors. While environmental air pollution will be measured using Radiello passive samplers and Gillian pumps. Data will be analyzed using the latest version of the STATANow/MP 19.5 software. Furthermore, health risk assessment will be conducted for lifetime non-carcinogenic and carcinogenic risk estimation following the USEPA framework. The study will identify environmental triggers that exacerbate asthma and other respiratory conditions in other similar community settings and will contribute to the body of knowledge in public health. Ethical approval was obtained from the Research Ethics Committee, Faculty of Health Sciences at the University of Johannesburg.

## 1. Introduction

Air pollution is associated with higher rates of childhood mortality and illness in low- and middle-income nations worldwide. Vulnerable groups such as senior populations, children and pregnant women, and those with chronic conditions are the most severely affected by air pollution [1]. Globally, it is estimated that air pollution causes ±9 million premature deaths per year, as well as the loss of millions of healthy years of life [2]. In Sub-Saharan Africa it has been linked to ±900,000 premature deaths [3]. The global burden of disease caused by ambient air pollution is well documented; however, data on the link between air pollution and negative health effects is still lacking in low- and middle-income countries in particular [1]. Yet, growing evidence shows that there is a high prevalence and severity of asthma, rhinoconjunctivitis, and eczema, which are much greater in areas with poor socio-economic conditions [4,5,6,7,8].

In 2015, asthma was identified as the most prevalent chronic respiratory disease globally [9]. Children are especially vulnerable to air pollution and are often exposed due to the time spent either indoors or outdoors [10]. Asthma, a long-term respiratory condition, is still widely recognized as a global public health problem, with epidemiological studies in recent decades showing an increase in related deaths globally [11,12]. Children living in informal settlements are more prone to develop asthma-like wheezing symptoms; however, asthma symptoms in children living in informal settlements still need to be identified, diagnosed, and treated [13]. There is evidence that ambient air pollution aggravates existing and pre-existing asthma and has the potential to cause oxidative injuries to airways, which in turn causes inflammation and increases the risk of sensitization [14]. Children in their preschool years are in their growth phase, which makes them vulnerable to air pollution and other contaminants [15]. Their research also suggested that the areas where air pollution was much higher showed a greater increase in respiratory diseases. The association between areas with high pollution and respiratory diseases in children was also seen in a study conducted in the Ekurhuleni Metropolitan Municipality, namely Tembisa and Kempton Park, with a study population of 3764 children [16].

A significant number of children attend early childhood centers for basic education. ECD centers are a place of care as the number of women who work has increased over the years. Solid fuel use, which is predominantly used for cooking, is highly prevalent in African and Southeast Asian countries and is a major contributor to indoor air pollution [17]. The results of a study showed that children aged 36 to 59 months exposed to solid fuels were 1.47 times more likely to have developmental delays compared with children who were not exposed to solid fuel use [18]. They may also have potential adverse effects on early childhood development. In another study, the researchers suggested there is an association between chemical and biological contaminants at daycare centers and wheezing in young children [19]. Interestingly, total volatile organic compounds (VOCs) and CO_2_ were the contaminants that were elevated in the results of the study, and VOCs were associated with reported wheezing in the questionnaire assessments [20]. Evidence shows that young children’s respiratory problems, particularly wheezing, were associated with exposure to poor indoor air quality and increasing levels of indoor air pollutants such as particulate matter (PM) and microbes [21,22]. Asthma may be more likely to develop among exposed children later in life due to indoor particle concentrations and the presence of bacteria in daycare centers [22]. A study in the United Arab Emirates found that nursery buildings’ CO_2_ and total VOC concentrations were 37% higher than World Health Organization (WHO) indoor air quality requirements, while the concentrations of total suspended particles (TSP) and formaldehyde (CH_2_O) were high as well [22,23]. It was also noted that the indoor pollutant concentration was higher than the outdoor air concentration.

Scientific evidence suggests that air pollutants play a significant role in the exacerbation of respiratory symptoms, with a bigger impact on children 5 years of age or younger. They are severely affected because of their undeveloped immune systems and respiratory systems, and this places them at a high risk of developing lung and other associated diseases. Furthermore, in highly congested areas such as informal settlements like Alexandra Township, pollution levels are high, leading to an impact on the respiratory disease burden. Several studies assessing exposure to PM_2_._5_ and benzene, toluene, ethylbenzene, and xylene (BTEX) pollutants have been conducted in South Africa [24,25,26]; however, not many studies focused on children under the age of 5 in ECD centers in a congested urban informal settlement. This is the first study to look at air pollution exposure in Alexandra, Gauteng province, South Africa. The results of this study will add to the body of knowledge on the subject matter in South Africa. This study aims to explore and describe the relationship between ambient air pollution and asthma-related conditions within a cross-sectional framework, focusing on the identification of patterns and correlations present in the data. The following objectives were set to achieve the aim of the study:

- To establish the association between asthma and related conditions and risk factors in the study population.
- To determine the impact of air pollutants on children under the age of 5 who attend ECD centers in township settings.
- To develop/design a model to address ambient air pollution and environmental risk factors contributing to the causation of asthma in informal settlements.

## 2. Materials and Methods

### 2.1. Study Design and Setting

The researcher will conduct a quantitative cross-sectional analytical survey. This design will assist in determining the prevalence of asthma and respiratory-related diseases and potential contributing risk factors among children aged 5 years or younger among the children attending ECD centers in Alexandra.

The study will be conducted in the Alexandra Township located in the City of Johannesburg, Gauteng Province. Due to rapid growth and urbanization in a short time, informal settlements, overcrowding, and high-rise buildings have become widespread in the city. The population size of the City of Johannesburg is 6,065,354. The population size of children under 5 living in Alexandra is 546,810. There are 162 ECD centers in Alexandra Township.

### 2.2. Study Population and Sampling

Children under the age of five years and their parents/caregivers are the target/study population, as the outcome of this study will be of benefit to future children in similar settings. Currently, there are approximately 162 identified early childhood development centers, with 6322 registered children, both registered and unregistered ECD centers. A list of ECD centers will be requested from the relevant authority. Each ECD center on the list will be allocated a unique number. A sample of ECD centers will be randomly selected and contacted by telephone to request permission to visit the ECD centers, where the purpose of the study will be explained. The study participants will be randomly selected using a simple random technique, where every second participant will be approached, and this will be repeated until the sample size has been met. The estimated study population is 6322; as mentioned earlier, a sample of 363 was calculated at a 95% confidence level, a 5% margin of error, and a 50% population proportion using Epi Info 7.2. However, the researchers will target a sample size between 1000 and 3000 to ensure a study power with an appropriate significance level as recommended in the ISAAC manual [27]. The proposed large sample size will enable the determination of statistical differences among the ECD centers in relation to the prevalence of asthma and other respiratory diseases when comparing ECD centers or groups of ECD centers during analysis.

Although the population to be studied includes the target group of children, the parents or caregivers will be in the best position to provide answers to the questionnaire, as they are aware of what the child has been exposed to. The study population will consist of all parents and their children (males and females) under the age of 5 attending early childhood development centers and their parents/caregivers in the township of Alexandra. In addition, children whose parents have given consent will be included in the study. The study will exclude children and parents/caregivers where consent is not acquired.

### 2.3. Data Collection Procedure

**Figure 1:**
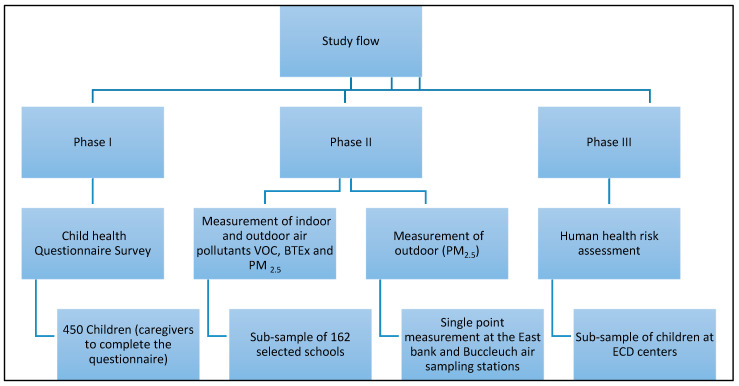
Schematic diagram: study flow of the proposed study.

The data collection was conducted in phases. Figure 1 shows the phases and the data collection tools to be used in each phase.

#### 2.3.1. Questionnaire: Child Survey to Assess the Health Outcomes

Phase 1 will use the International Study of Asthma and Allergies in Childhood (ISAAC) modified questionnaire (see Supplementary File S1) to address the prevalence of respiratory disorders and potential risk factors in children under the age of 5 years [27,28,29]. The International Study of Asthma and Allergies in Childhood, or ISAAC, was an innovative global epidemiological research initiative that was started in 1991 to study childhood asthma, rhinitis, and eczema in response to growing concerns that these conditions were becoming more prevalent in both developed and developing nations, including South Africa [29,30,31,32,33]. While the questionnaire was initially designed for children older than five years, this study will provide an opportunity to validate and test the reproducibility of the ISAAC questionnaire when administered to the population under five years. The researchers anticipate that the questionnaire will be suitable for this study population because it has been tested in children aged 6–7 and 13–14 years, and a similar study was conducted in the Mpumalanga province of South Africa within a comparable age group (preschoolers) [33,34,35].

The proposed research project will utilize a structured, paper-based, interviewer-administered questionnaire to gather data, with parents responding to the questionnaire. The questionnaire will consist of four sections, viz., sociodemographic, respiratory symptoms, household exposures, and ECD center environmental exposures. The questionnaire will be reviewed and approved by the supervisor and a biostatistician before the study commences. Language barriers are not anticipated, as the questionnaire will be translated into seSotho and isiZulu. The researcher will appoint an experienced research assistant to participate in the data collection. The questionnaire will be administered face-to-face and take approximately twenty minutes to complete. The time activity pattern will be kept with the parent and ECD centers for two weeks for completion.

#### 2.3.2. Environmental Sampling

Environmental sampling will be conducted to achieve objective two, which will entail the measurement of indoor pollutants such as VOCs and BTEX, as well as outdoor PM_2.5_. Firstly, the indoor and outdoor VOCs and BTEX will be measured using Radiello passive samplers over a 14-day (two-week) period at the 162 selected ECD centers by the researcher and two field workers. The sampling will be conducted at the end of autumn and the beginning of the winter seasons to determine seasonal and temporal variation in pollutants during the two most polluted seasons [36,37]. One classroom will be selected from each of the selected ECD centers to deploy the samplers. The Radiello sampling system primarily consists of a diffuse body, supporting plate, vertical adapter, and a collection cartridge in a sealed glass tube. In addition, an adhesive label with the barcode indication will be included. For each gaseous component, there is a specific collection cartridge and protocol to be followed. The indoor passive samplers will be positioned at a height of approximately 1 m above ground surfaces to avoid contamination and to keep them out of the sight of children to preventing tampering. The outdoor samplers will be placed within a protective shelter to protect them from extreme weather conditions such as sunlight, strong winds, and rain; however, the shelter will still allow for adequate airflow.

Thereafter, PM_2.5_ sampling will be conducted using small Gillian pumps over 24 h for two weeks. The pumps will be calibrated according to factory standard procedure before sampling. At the sampling site, the sampling line will be completed by linking the filter holder to a calibrated pump. To separate the respirable fraction of airborne dust from the total dust, a cyclone separator will be used as part of the filter/cassette holder to be assembled at a flow rate of 4 L/min. The outdoor PM_2.5_ measurements will be performed on the rooftop of the ECD centers. The PM_2.5_ pumps will be placed in plastic containers for protection. The pumps will be switched on and the time recorded. At the end of the sampling period, the pumps will be switched off, and the time will be recorded. The researcher and an assistant will conduct all the gravimetric analyses of the PM_2.5_ filters at an accredited laboratory.

### 2.4. Human Health Risk Assessment (HHRA)

Phase 3 will identify the potential future harm due to VOC and BET exposure from an early age using an HHRA for the children who attend ECD centers in the area. We need to determine the amount of time children spend indoors and outdoors, their exertion levels, and exposure concentration. A diary for seven days will be kept for the sample children. The time activity pattern will be completed by the teachers of the ECD center as well as the parents after the ECD center hours and on weekends. The diary will be distributed by the researcher to the teachers of the ECD center, and the teachers will deliver it to the parents.

The lifetime adjusted daily intake [LADI] will be used to determine the assessment calculation. The lifetime adjusted daily intake for both carcinogenic (benzene) and non-carcinogenic (toluene, ethylbenzene, and xylene) risks will be calculated using Equation (1).

LADI (*averaged daily intake*) = (C × CF × IR × YE × ED)/(BW × AT) (1)

where C is exposure concentration (µg/m^3^); IR = inhalation rate (m^3^/day); ET = exposure time (hours/day); EF = exposure frequency (days/year); ED = exposure duration (years) to be calculated using Equation (2); BW = body weight (kg); and AT = averaging time (days). See Table 1 for a description of the parameters.

ED = (Actual exposure duration)/(24 h) × 92 days(2)

Thereafter, we determine the cumulative lifetime exposure concentration intake. Using Equation (2), the lifetime average chronic intake was calculated for 30 years [LADIadj] based on the USEPA default value: Where LADI_[adj]_ is the cumulative average 30-year intake dose [mg/kg/day]; LADI is the chronic daily intake [mg/kg]; EF is the estimated lifetime exposure duration, which is equivalent to 30 years; 365 is the total number of days in a year; and the life expectancy is estimated to be 61.5 and 67.7 years for both males and females, respectively, in South Africa.

LADI_[30 years dose]_ = ∑CDI × 365 × YE (3)

The reference levels in the calculation below (Equation (4)) will be used as a benchmark for the risk probability determination.

CR = LADI_[adj]_ × SF (4)

where SF is the slope factor for carcinogenic pollutants [0.0273]; CR refers to the carcinogenic risk; and LADI_[adj]_. The cumulative lifetime adjusted intake is calculated using Equation (2) mentioned above, where the lifetime estimated exposure for 60 years will be determined.

The hazard quotient [HQ] will be used to estimate the potential non-carcinogenic risk. If the HQ value is greater than 1, it will be regarded that BTEX pollutants have a potential of causing non-carcinogenic effects, while an HQ value of less than 1 means there is a low probability of developing associated health effects. The procedure for calculating HQ is shown in Equation (4).

HQ = LADI_[adj]_/reference Concentration (RfC) (5)

where HQ is the hazard quotient; LADI_[adj]_ is the cumulative average intake dose; and RfC is the reference.

### 2.5. Quality Control

A pilot test will be conducted at one of the ECD centers. The participants involved in the pilot study will not be included in the actual study. The questionnaire will be designed to measure the risk factors in home and ECD environments that predispose a child to asthma and related conditions. It will be designed based on the ISAAC questionnaire, which has been used widely in other countries. All questions in the questionnaire will be relevant to the research question. The key aim of the pilot study is to ensure the accuracy and appropriateness of the questionnaire within the targeted population.

To ensure quality control, the samplers will be retained within their original containers at room temperature before sampling. During transportation, they will be maintained in cooler boxes to ensure an adequate temperature is upheld. The samplers will be transported to an accredited laboratory immediately following their removal from the site of exposure assessment. Throughout this process, a chain of custody will be maintained to prevent any potential tampering before laboratory analysis. Additionally, a field blank sample will be retained from each batch of samplers and submitted to the laboratory for analysis.

### 2.6. Data Analysis

The primary data will be recorded, cleaned, coded, and captured into Microsoft Excel, which will then be exported to the latest version of STATANow/MP 19.5 software for analysis. The categorical variable will be presented using frequency distribution tables and figures. The continuous variable will be reported using measures of central tendency (such as mean with standard deviation, median, and range) and box plots where applicable. The *t*-test and chi-square test will be used to determine statistical significance for continuous and categorical variables, respectively.

To identify the risk factors associated with asthma in this study, a multiple logistic regression model will be employed, incorporating odds ratios (ORs) and 95% confidence intervals (CIs). Initially, a bivariate binary logistic regression analysis will be performed to assess the relationship between the dependent variables (asthma and other respiratory conditions) and various socio-demographic, environmental, household, and early childhood development (ECD) risk factors, presented with crude odds ratios and 95% CI. Household conditions that may contribute to exposure include the type of residence (formal or informal), ownership of pets or animals, exposure to environmental tobacco smoke in the home, and the type of fuel utilized for cooking and heating. Additionally, potential exposure and confounding factors will encompass the duration of residence in the area (less than 3 years versus 3 years or more), the location of the home and ECD facility, proximity to major thoroughfares, the health status and dietary history of the child, as well as the parents’ knowledge and perceptions regarding air pollution and its health implications. In the final model, a backwards stepwise selection method based on the Wald test will be applied within the multiple logistic regression analysis to ascertain the risk factors associated with asthma and other respiratory health outcomes, while controlling confounding variables. Adjusted odds ratios (AORs) with 95% CIs will be calculated to evaluate the likelihood of associations between asthma, other respiratory outcomes, and the identified risk factors. Lastly, statistical significance will be established with a *p*-value threshold set at 0.05.

### 2.7. Data Management and Ethics

Data collected in the study will be accessed by the research team (researcher and supervisors). Data will be stored on the e-drive with a password encrypted, while the hard copies will be stored in a lock and key storage. Data will be kept for ten years on the University of Johannesburg server and thereafter archived. Confidentiality will be maintained and compliance with the POPIA Act. The information from the study will not be shared with anyone else, unless it is required by law, and the request must be submitted according to the POPIA Act. Permission will be requested from UJ for data sharing for other studies. The statistical analyses will be conducted by the applicant and checked by the statistician from the University of Johannesburg.

Permission to conduct this study was obtained from the Gauteng Department of Education, the jurisdiction of the ECD centers. This study received ethical clearance from the Faculty of Health Sciences Research Ethics Committee (REC) of the University of Johannesburg (REC-2662-2024) and approval from the Higher Degrees Committee at the University of Johannesburg (HDC-01-03-2024). Prior to this study commencing, parents/guardians of the children signed consent and assent forms, respectively, and were given the option to withdraw from the study.

## 3. Discussion

The WHO estimates that air pollution is responsible for 3.1 million premature deaths worldwide every year, which accounts for 3.2% of the global burden of disease [38]. In addition, low- and middle-income nations in Southeast Asia and the Western Pacific regions had the biggest impact on the air pollution burden in 2012, with 7.1 million deaths, while Africa had more than 600,000 deaths [39]. The quality of air that communities breathe is a critical social determinant and is often responsible for the disproportionate disease burden in high socioeconomic deprivation areas [40]. Alexandra Township is a previously disadvantaged area that has a myriad of social, economic, and environmental challenges (including air pollution), which is a contrast to its neighboring residential area, the élite Sandton. Yet, there is limited evidence and research studies on the health impacts of air pollutants in vulnerable and exposed populations such as Alexandra.

**Figure 2:**
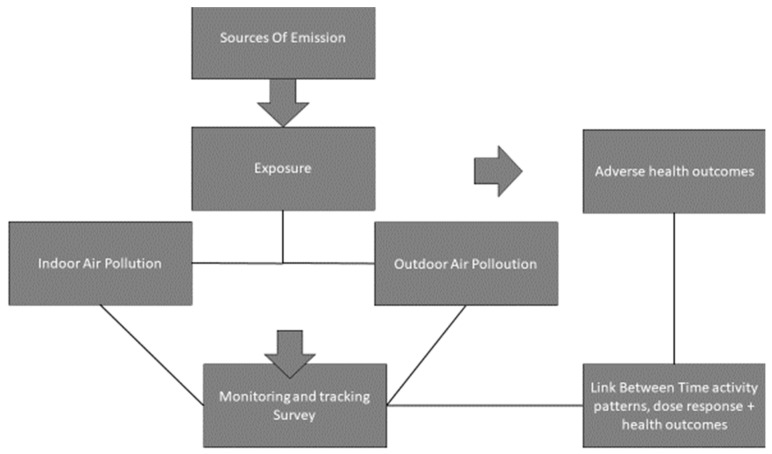
The study’s conceptual framework illustrates the link between children’s exposure to air pollution and their respiratory outcomes.

The identification and assessment of these dimensions lead to responsive actions among relevant stakeholders. A conceptual framework (Figure 2) was developed to demonstrate the connections between children’s exposure to air pollution and the monitoring of specific pollutants. It defines the study’s objectives and the key factors, concepts, and variables involved. Supporting evidence from the literature review indicates that air pollution poses a health threat or risk. The framework is presented diagrammatically to depict the presumed relationship between air pollutants and childhood respiratory diseases. Therefore, the results of this study will enhance the body of knowledge in public health and guide policymakers and implementers. The research will evaluate, refine, and validate new scientific findings in public health. A model will be developed to assist environmental health practitioners at the operational level in implementing preventive measures to address the impact of ambient air pollution on asthma. This will contribute to improving the quality of life for the population and in other similar community settings.

### Strengths and Limitations

One of the strengths of this study is its utilization of both internationally and locally validated ISAAC questionnaires, which have been translated into two local languages. Furthermore, the study will assess air quality in both indoor and outdoor environments to quantify the levels of air pollutants in early childhood development centers, where children spend the majority of their day.

The anticipated limitations of the study include, firstly, its reliance on a cross-sectional study design, which is effective for assessing environmental factors and health outcomes at a singular point in time; however, this design has a limited capacity to establish causal relationships. Additionally, the environmental monitoring of air pollutants will be conducted during the autumn and winter seasons, which are reported to be the most polluted periods of the year. Consequently, the results may not be generalizable across the entire year and should be interpreted as indicative of a worst-case scenario.

## 4. Conclusions

According to both scientific and anecdotal evidence, there is a scarcity of environmental health strategies aimed at mitigating the effects of exposure to ambient pollutants—such as particulate matter, volatile organic compounds (VOCs), and benzene, toluene, ethylbenzene, and xylene (BTEX)—on respiratory illnesses among toddlers residing in disadvantaged, low-to-middle-income communities. Moreover, there is a notable absence of environmental epidemiology research that addresses this issue in townships such as Alexandra. Consequently, a cross-sectional analytical study employing various methodologies, including an internationally validated questionnaire and environmental measurements, has been designed to investigate this phenomenon within the Alexandra township. Environmental asthma is not classified as a reportable condition in South Africa. Consequently, the findings of this study aim to inform child health policy by emphasizing the significance of early screening for asthma and respiratory diseases, as well as the necessity for environmental monitoring, particularly concerning harmful pollutants in children’s environments. Furthermore, this research will enhance the existing body of knowledge in public health, specifically within the domain of environmental epidemiology. The study will also underscore the importance of routine data collection on pediatric air pollution exposure as part of a proactive health strategy for managing and controlling environmental asthma (and related respiratory illnesses) and its environmental factors. A model will be developed to assist environmental health practitioners (EHPs) at the operational level in implementing preventive measures that address the impact of ambient air pollution on asthma. Ultimately, this initiative aims to improve the quality of life for the population and in similar community contexts.

## Figures and Tables

**Table 1:** The description of exposure parameters and their measurement units.

Parameter	Description	Unit
C	Exposure Concentration	µg/m^3^
IR	Inhalation	m^3^/day
BW	Body Weight	kg
ED	Exposure days	Days/year
YE	Estimated future years of exposure	Years
AT	Years in a lifetime	Years

## Data Availability

There is no data available at this stage, as new data has not been created and analysed yet.

## Supplementary Materials

The following supporting information can be downloaded at: https://www.mdpi.com/article/10.3390/mps8040084/s1, File S1: The questionnaire
