# Supplementary material for: A Study Protocol to Assess the Association Between Ambient Air Pollution and Asthma and Other Respiratory Health Outcomes Amongst Children Below 5 Years of Age in Alexandra Township’s Early Childhood Development Centers, Johannesburg
Source: Methods Protoc. 2025 Aug 1;8(4):84. doi: 10.3390/mps8040084 (PMC12388445; doi:10.3390/mps8040084)
Supplement: Supplementary file 1 [file mps-08-00084-s001.zip › mps-3670835-supplementary.pdf]

<u>Supplementary File 1 (SF1): The questionnaire</u>

International Study of Asthma and Allergies in Childhood (ISAAC)

Questionnaire

Instructions for completing the questionnaire

Below are some examples of questionnaire completion instructions.

On this sheet are questions about your child's name, ECD, and age. Please write your answers to these questions in the space provided.

All other questions require you to tick your answer in a box. If you make a mistake put a cross in the box and tick the correct answer. Tick only one option unless otherwise instructed.

Examples of how to mark questionnaires: Age [4] years

To answer Yes/No, put a cross in the appropriate box as per the example below:

YES	
NO	X

SOCIODEMOGRAPHIC SECTION

SUBURB/ TOWNSHIP/SECTION WHERE YOUR CHILD LIVES: [ ]

YOUR CHILD'S SCHOOL: [ ]

TODAY'S DATE: [ ]

YOUR CHILD'S AGE: [ ] years

WEIGHT OF YOUR CHILD? ☐ Kg

HEIGHT OF YOUR CHILD? ☐ Metres /centimetres (Please circle the measurement you used)

RESPIRATORY SYMPTOMS SECTION

(Tick all your answers for the rest of the questionnaire)

Is your child a: ☐ **MALE** ☐ or **FEMALE?**

Core questionnaire for asthma

1. Has your child ever had wheezing or whistling in the chest at any time in the past?

Yes	
No	

2. Do you or a family member have a history of asthma or allergies

Yes	
No	

IF YOU HAVE ANSWERED "NO" PLEASE SKIP TO QUESTION 6

3. How many attacks of wheezing has your child had in the past 12 months?

None	
1 TO 3	
4-14	
More than 12	

4. In the past 12 months, how often, on average, has your child's sleep been disturbed due to wheezing?

Never woken with wheezing	
Less than one night per week	
One or more nights per week	

5. In the past 12 months, has wheezing ever been severe enough to limit your child's speech to only one or two words at a time between breaths?

Yes	
No	

→ 6. Has your child ever had asthma?

Yes	
No	

7. Was the asthma diagnosed by a medical doctor or nurse?

Yes	
No	

8. In the past 12 months, has your child's chest ever sounded wheezy during or after playing?

Yes	
No	

9. In the past 12 months, has your child had a dry cough at night, apart from a cough associated with a cold or chest infection?

Yes	
No	

Core questionnaire for rhinitis

10. Has your child ever had a problem with sneezing or a runny, or blocked nose when she/he DID NOT have a cold or the flu?

Yes	
No	

IF YOU HAVE ANSWERED "NO" PLEASE SKIP TO QUESTION 14

11. In the past 12 months, has your child had a problem with sneezing, or a runny, or blocked nose when she/he DID NOT have a cold or the flu?

Yes	
No	

IF YOU HAVE ANSWERED "NO" PLEASE SKIP TO QUESTION 14

12. In the past 12 months, has this nose problem been accompanied by itchy-watery eyes?

Yes	
No	

13. In which of the past 12 months did this nose problem occur? (Please tick any which apply)

January		July	
February		August	
March		September	
April		October	
May		November	
June		December	

14. In the past 12 months, how much did this nose problem interfere with your child's daily activities?

Not at all	
A little	
A moderate amount	

A lot	

15. Has your child ever had hay fever?

Yes	
No	

16. Was the hay fever diagnosed by a doctor or nurse?

Yes	
No	

Core questionnaire for eczema

17. Has your child ever had an itchy rash, which was coming and going for at least six months?

Yes	
No	

IF YOU HAVE ANSWERED "NO" PLEASE SKIP TO QUESTION 21

18. Has your child had this itchy rash at any time in the past 12 months?

Yes	
No	

IF YOU HAVE ANSWERED "NO" PLEASE SKIP TO QUESTION 21

19. Has this itchy rash at any time affected your child in any of the following places:

Folds of elbows	
Behind knee	
Front of ankles	
Under buttocks	

Around eyes or ears	

20. At what age of the child did this itchy rash first occur?

Age 2-4 years	
Age 5 or more	
Can't remember	

21. Has this rash cleared completely at any time during the past 12 months?

Yes	
No	

22. In the past 12 months, how often, on average, has your child been kept awake at night by this itchy rash?

Never in the past 12 months	
Less than one night per week	
One or more nights per week	

23. Has your child ever had eczema?

Yes	
No	

24. Was the eczema diagnosed by a doctor or nurse?

Yes	
No	

HOUSEHOLD EXPOSURE SECTION

1. How long has your child lived in this SUBURB/ TOWNSHIP/SECTION?

Less than 6 months	
6 to 12 months	
1 to 2 years	
3 years or longer	

2. How does your child usually get to school?

Walk	
Taxi/Bus	
Motor car	
Combination	
Other	

3. How far is the nearest Clinic or Hospital from your home?

15-minute walk or 5-minute drive	
1-hour walk or 15-minute drive	
more than an hour's walk or more than 30-minute drive	

4. What type of house does your child live in?

Brick	
Mud	
Corrugated iron	
Combination	
Other	

5. How many rooms are in your house? (Excluding bathrooms)

6. Do you have running water in the house?

Yes	
No	

7. In the past 12 months, how often, on average, did your child eat or drink the following?

Type of food	Never or occasionally	Once or twice per week	Three or more times per week
Meat (e.g. beef, lamb, chicken, pork)			
Seafood (including fish)			
Fruit			
Vegetables (green and root)			
Pulses (peas, beans, lentils)			
Cereal (including bread)			
Pasta			
Rice			
Butter			
Margarine			
Nuts			
Potatoes			
Milk			
Eggs			
Fast food/burgers			

8. In the past 6 months, how often has your child been absent from school?

Never or occasionally	
Once or twice per week	
Three or more times a week	

9. In your house, what fuel is usually used for cooking?

Electricity	
Gas	
Paraffin	
Open fires	
Other – Please specify	

10. In your house, what fuel is usually used for heating?

Electricity	
Gas	
Paraffin	
Open fires (wood, coal)	
Other – Please specify	

11. In the past 12 months, how often, on average, have you given your child Salbutamol, asthavent or other asthma medication?

Never	
At least once a year	
At least once per month	

12. Was your child born in this township/suburb?

Yes	
No	

16. How often do trucks pass through the street where you live, on weekdays?

Never	
Seldom	
Frequently throughout the day	

Almost all-day	

17. Do you currently have a cat in your home?

YES	
NO	

18. In the past 12 months, have you had a cat in your home?

YES	
NO	

19. Do you currently have a dog in your home?

YES	
NO	

20. In the past 12 months, have you had a dog in your home?

YES	
NO	

21. Does the child's mother (or female guardian) smoke cigarettes?

YES	
NO	

22. Does the child's father (or male guardian) smoke cigarettes?

YES	
NO	

23. How many people living in the same house as your child smoke cigarettes?

	People

24. How would you rate the indoor air quality in your home?

Good	
Average	
Poor	

ENVIRONMENTAL EXPOSURE SECTION

1. How would you rate the outdoor air quality in your community?

Good	
Average	
Poor	

2. **Please express how much you agree or disagree with the following statements**

	**Strongly agree**	**Agre e**	**Undecided**	**Disagree**	**Strongly disagree**
Improving the environment is the responsibility of every citizen					
Recycling programs should be put in place and promoted across the whole city					
I am actively involved in cleaning up the environment					
The pollution is out of my control and I cannot do anything to change it					

If I knew how to better contribute to a cleaner environment, I would take action					
I do not see the pollution as a health problem					

THANK YOU
